# Selective arterial embolization of renal angiomyolipomas: A 10‐year experience

**DOI:** 10.1002/bco2.107

**Published:** 2021-08-31

**Authors:** Guram Nozadze, Signe Benzon Larsen, Søren Heerwagen, Ruben Juhl Jensen, Lars Lönn, Martin Andreas Røder

**Affiliations:** ^1^ Department of Urology, Urological Research Unit Copenhagen University Hospital – Rigshospitalet Copenhagen Denmark; ^2^ Department of Clinical Medicine University of Copenhagen Copenhagen Denmark; ^3^ Department of Radiology Copenhagen University Hospital – Rigshospitalet Copenhagen Denmark

**Keywords:** efficacy, eGFR, renal angiomyolipoma, safety, selective endovascular trans‐arterial embolization

## Abstract

**Objectives:**

To study safety and efficacy of selective endovascular trans‐arterial embolization (TAE) of renal angiomyolipoma (AML) in a 10‐year period at a regional tertiary referral center in Denmark.

**Patients and methods:**

All 56 patients who underwent TAE of renal AML at Departments of Urology and Radiology, Copenhagen University Hospital – Rigshospitalet, Denmark, from 2009 to 2020 were included. Seven without preoperative and postoperative imaging were excluded, leaving 49 patients for analysis. From national electronic medical records, we retrieved patient characteristics, surgical data, and follow‐up data. Tumor size at the time of embolization and during follow‐up was compared using Student's paired *t* test. Estimated glomerular filtration rate (eGFR) pre‐ and post‐embolization were compared using Wilcoxon rank sum test.

**Results:**

We included 49 patients of whom 4 had two tumors treated in the same TAE procedure. Median age was 50 years (interquartile range [IQR]: [29–67 years]), and the median follow‐up time was 4.6 years [IQR: 3.0–6.7 years]. Post‐embolization syndrome (PES) was experienced in 27 patients, and non‐PES in 5 patients. Median length of hospital stay was 0 days [IQR, 0–1]. Postoperative Everolimus immunosuppressive treatment was offered to seven patients. Median tumor size was 6.0 cm [IQR: 4.6–7.9 cm] and was significantly reduced to 3.7 cm [IQR: 2.5–5.2 cm] after treatment (*p* < 0.001). Kidney function was not affected by TAE. Three deaths, not related to AML, were noted during follow‐up.

**Conclusion:**

Embolization of AML was in this cohort effective to significantly reduce tumor size without serious adverse events and loss of renal function. TAE is a safe and efficacious treatment and the preferred minimally invasive treatment option of AML.

## 1 INTRODUCTION

Angiomyolipoma (AML) is an uncommon benign renal mesenchymal tumor with female dominance and a prevalence of up to 0.8%. Histologically, it is a mixture of blood vessels, smooth muscle‐like cells, and adipose tissue.^1^ AML can be diagnosed with ultrasound, computed tomography (CT), or magnetic resonance imaging (MRI), but ultrasound carries a risk of poor specificity for excluding renal cell carcinoma (RCC) as a differential diagnosis.^2^ Approximately 80% of AMLs occur sporadically, the remaining due to tuberous sclerosis complex (TSC) or lymphangiomyelomatosis (LAM), both rare genetic conditions.^3^, ^4^, ^5^

Due to tortuous aneurysmatic arteries, AML is prone to rupture spontaneously and cause pain, hematuria, retroperitoneal hemorrhage, or even death. Although benign in nature, AML is a concern in pregnancy as well as if lesions are >4 cm where profylactic treatment is preferred due to the risk of rupture.^6^ In patients with TSC, AML can be treated with a mammalian target of rapamycin (mTOR) inhibitor (Everolimus).^7^ Traditional surgical options include open or laparoscopic removal of the tumor using nephron‐sparing surgery or total nephrectomy, the latter in large, complicated tumors. Minimal invasive treatments include thermal ablation or selective endovascular trans‐arterial embolization (TAE). Prophylactic surgery reduces the risk of rupture but must carry limited side effects due to the benign nature of the disease. The most frequent treatment of AML is abdominal surgery with its inherent risk of postoperative complications.^8^

Selective TAE of renal AML is a minimally invasive treatment with few complications and a tempting option because it is a more nephron‐sparing option than surgery.^9^ Studies are still limited with short follow‐up.^10^, ^11^, ^12^, ^13^, ^14^ This retrospective single‐center study presents the safety and efficacy on planned and acute selective TAE treatment of AML over a 10‐year period at our tertiary referral center.

## 2 PATIENTS AND METHODS

This study included all patients who underwent prophylactic or emergency treatment with TAE of AMLs at the Departments of Urology and Radiology, Copenhagen University Hospital – Rigshospitalet, Denmark, from August 3, 2009 to June 29, 2020. According to the local protocol, prophylactic TAE is advised for asymptomatic AML ≥ 4 cm, and as an emergency or subacute procedure if the patient shows signs of active bleeding with flank pain and hemodynamical instability. In all, 56 patients were identified of whom seven without preoperative and postoperative imaging were excluded, leaving 49 patients for analysis. National electronic medical records were used to retrieve all data until October 2020. Radiological and clinical follow‐up time was defined as the time from the embolization procedure, until October 1, 2020. This study received approval from The Danish Patient Safety Authority (case number: 31‐1521‐405) and the Capital Region of Denmark (P‐2020‐654).

Among the 49 included patients, we included only their first occasions of TAE procedure. However, in four patients, two tumors were embolized during the same procedure, resulting in a total of 53 tumors being embolized in 49 patients.

Body mass index (BMI) was grouped into three categories: <25, 25–30, and >30, respectively. Physical status was classified using the American Society of Anesthesiologists (ASA) system.^15^ Symptoms at presentation were recorded from the embolization procedures of the 49 patients. Post‐embolization syndrome (PES) was defined as fever (above 38.0°C), nausea, and abdominal pain.^16^ Other complications were defined as non‐PES complications.

Tumor size before and after embolization was assessed by an experienced radiologist. The final tumor size after embolization was recorded from the latest available imaging until October 2020. Not all tumors had three‐dimensional recordings and thus the maximal diameter on axial CT/MR scans was used to record tumor size. Tumors were measured various times throughout the follow‐up time. The national laboratory database was reviewed for estimated glomerular filtration rate (eGFR). Preoperative blood tests were recorded as the latest tests available before TAE. Postoperative blood tests were recorded as the latest logged before discharge. If the blood sample was not reported within 2 weeks following TAE, it was regarded as missing.

**FIGURE 1:**
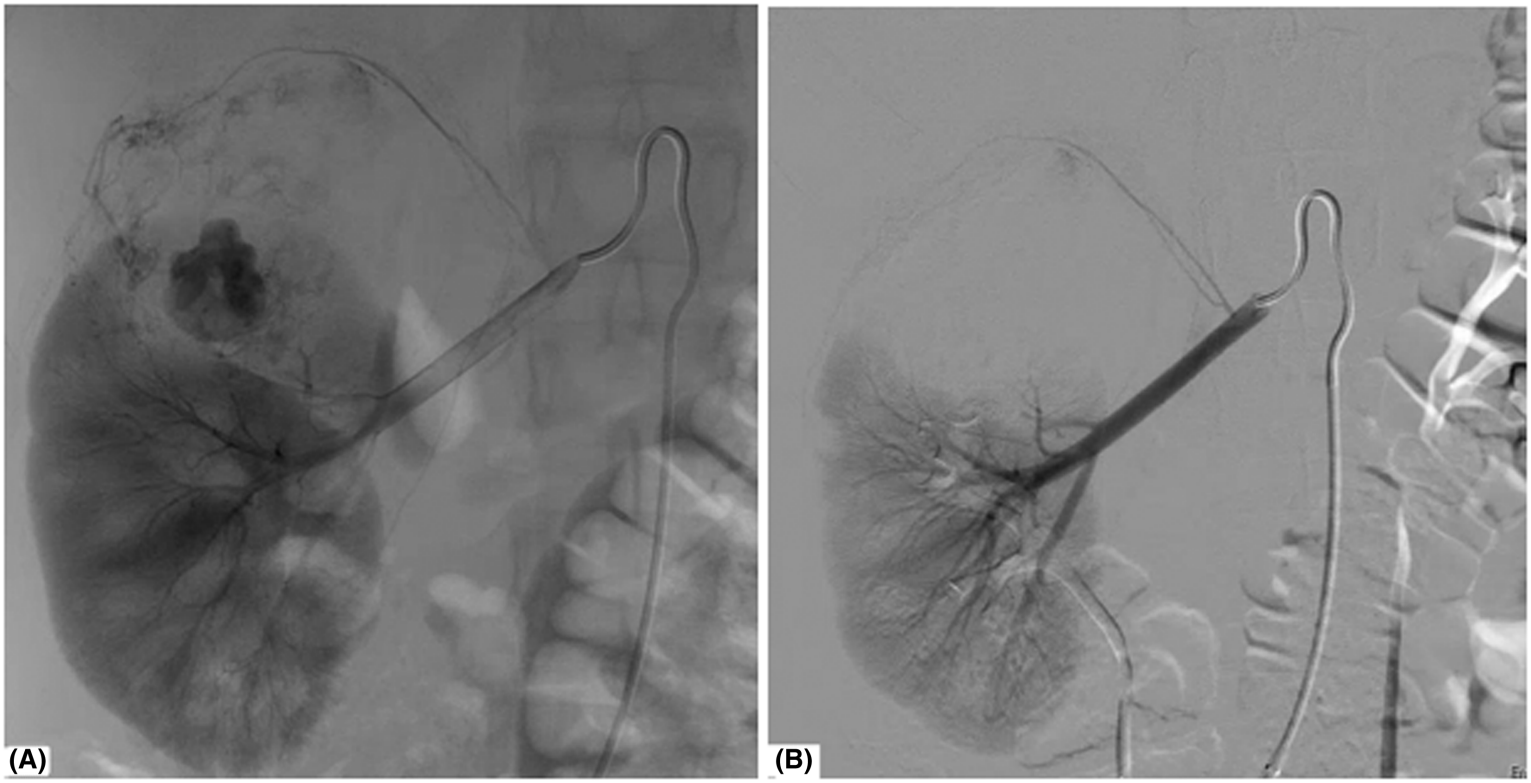
(A) Pre‐embolization image of a kidney (renal artery and aneurism filled with contrast). (B) Post‐embolization image of the same kidney filled with nonspherical polyvinyl alcohol (nsPVA) 355–500 μm and microspheres 250 μm (Embozene®, Boston Scientific)

TAE was performed via the common femoral artery in local or general anesthesia. Selective renal arteriography was performed using a 4 or 5 Fr. diagnostic catheter followed by super‐selective catheterization and embolization of the feeding artery (ies) using a microcatheter. Embolization was performed using particulate agents: nonspherical polyvinyl alcohol (nsPVA) 355–500 μm (Bearing nsPVA®, Merit Medical) or microspheres 250 μm (Embozene®, Boston Scientific), Figure 1. In selected cases, one or more microcoils (Tornado® or Nester®, both Cook Medical) were placed. Most often, this was used in combination with particles in the treatment of microaneurysms. Puncture site hemostasis was obtained using an Angio‐Seal® (Terumo) and/or manual compression.

## 3 STATISTICAL ANALYSES

Hospitalization days, follow‐up time, tumor size, and age were reported as medians with interquartile range (IQR). Nominal variables were reported as counts and percentages. Tumor sizes at the time of embolization and at the end of follow‐up were compared using Student's paired *t* test. eGFR pre‐ and post‐embolization were compared using Wilcoxon rank sum test. A *p* value of <0.05 was considered statistically significant. Changes in tumor size and changes in the weighted median size in the study period were plotted by linear regression and by the LOESS method. Data analysis was performed in R (version R‐3.6.1).

## 4 RESULTS

Of the 49 patients, 39 were females (Table 1). The median age was 50 years [29–67 years] at the time of diagnosis. The median follow‐up time was 4.6 years [IQR, 3.0 6.7 years]. A total of 20 patients were diagnosed with either TSC, LAM, or both. AML was detected incidentally in 36 patients. Only 13 patients had clinical symptoms prior to imaging diagnosis, and 22 patients had bilateral tumors; however, only 4 patients received treatment for two tumors in the same procedure.

**TABLE 1:** Characteristics of 49 patients with AML treated with embolization from August 3, 2009 to June 29, 2020

Patient characteristics	Number (%)
Sex	
Female	39 (79.6)
Male	10 (20.4)
Age	
Years, median [IQR]	50 [29–67 years]
BMI	
<25	23 (46.9)
25–30	17 (34.7)
>30	6 (12.3)
Missing	3 (6.1)
ASA score	
ASA1	14 (28.6)
ASA2	18 (36.7)
ASA3/ASA4	17 (34.7)
Underlying disease	
Tuberous sclerosis or lymphangioleiomyomatosis	20 (40.8)
AML Detection	
Symptomatic patient CT	13 (26.5)
Incidental finding (CT/MR/Ultrasound)	36 (73.5)
Laterality	
Bilateral	22 (44.9)
Unilateral	27 (55.1)
Tumor size before embolization, cm, median [IQR]	6.0 [4.6–7.0]
Intralesional aneurism > 5 mm	5 (10.2)
Symptoms and objective findings at presentation prior to first embolization^a^	
Yes	28 (57)

Abbreviations: AML, angiomyolipoma; ASA, American Society of Anaesthesiologists; BMI, body mass index; IQR, interquartile range.

^a^ Symptoms and objective findings included flank pain, retroperitoneal hemorrhage, hypotension, macroscopic hematuria, microscopic hematuria, and palpable mass.

Out of the 49 patients, 39 had prophylactic treatments of AML, and 10 were acute/subacute (Table 2). The median perioperative time was 60 min [IQR: 51–62 min]; however, in 26 patients, the procedure time was not noted in the x‐ray protocol. Local anesthesia was used in 44 patients with no perioperative blood loss. In addition to nsPVA or microspheres, microcoils were used as a supplement in 10 procedures. Intralesional aneurism >5 mm was identified in connection with five procedures. Angio‐Seal® (Terumo, Europe) vascular closure device was deployed in the femoral common artery for hemostasis, and some patients also needed manual compression.

**TABLE 2:** Perioperative characteristics of 49 patients from August 3, 2009 to June 29, 2020

Perioperative characteristics	Number (%)
Operation type	
Acute/semi‐acute	10 (20.4)
Prophylactic	39 (79.6)
Anesthesia	
General anesthesia	5 (10.2)
Local anesthesia	44 (89.8)
Vascular closure	
Manual compression	9 (18.4)
Angioseal	40 (81.6)
Embolizing material	
Coiling with Tornado or Nestar (+PVA)	10 (20.4)
PVA particles or Embozene	39 (79.6)

Abbreviation: PVA, polyvinyl alcohol particles.

The median length of stay was 0 days [IQR: 0–1] (Table 3). Postoperative antibiotic treatment was prescribed to eight patients, and postoperative nonsteroid anti‐inflammatory drugs (NSAID) treatment in 26 patients, which mirrors the PES diagnosis recorded in 27 procedures. Only one patient was readmitted within 30 days after the TAE procedure, due to PES complications. Non‐PES complications were observed in five patients, which included two closure device failures, one kidney abscess, one pseudoaneurysm formation, and one patient treated for pneumonia right after embolization.

**TABLE 3:** Postoperative characteristics of 49 patients treated with embolization from August 3, 2009 to June 29, 2020

Postoperative characteristics	Number (%)
Hospitalization	
Days, median [IQR]	0 [0, 1]
Postoperative treatment	
NSAID	25 (51.0)
Antibiotics	8 (16.3)
Complications^a^	
Post‐embolization syndrome (PES)	27 (55.1)
Non‐PSE complications	5 (10.2)
No‐complications	17 (34.7)

Abbreviation: NSAID, nonsteroid anti‐inflammatory drugs.

^a^ Recorded during 30 days of post‐embolization.

Ten patients were re‐embolized during the follow‐up time due to incomplete response or increasing size of AML. Postoperative Everolimus treatment was offered to seven patients after embolization. The dose was not noted, but compliance problems due to side‐effects were reported.

**FIGURE 2:**
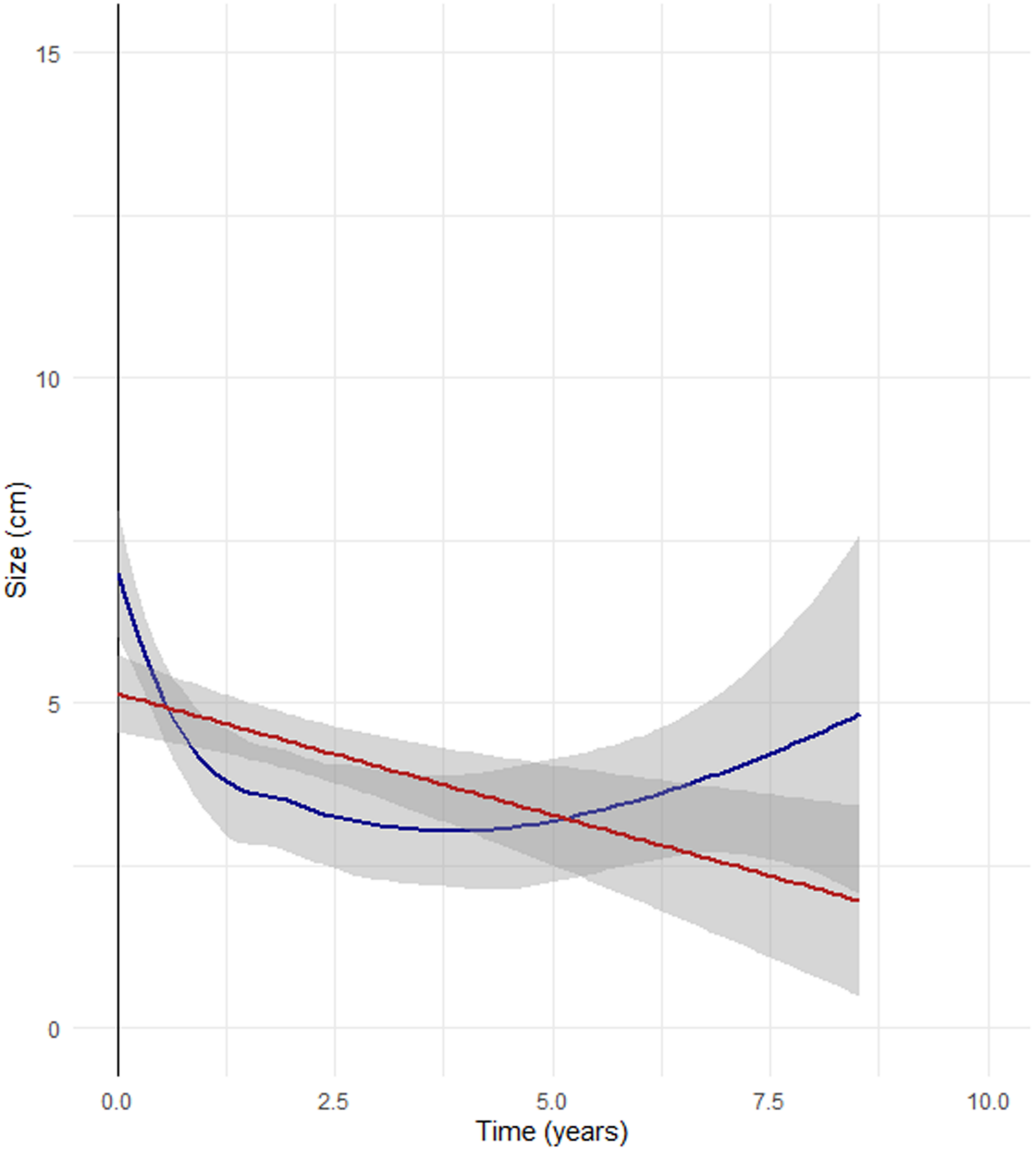
The change in tumor (*n* = 53) size and change in the weighted mean size in the study period. Tumors were measured various times throughout the follow‐up time. *X*‐axis: time in years; *Y*‐axis: size in cm; *Y*‐intercept (Time 0) represents the time of the embolization. Straight‐line (red): linear graph of tumor reduction; curved line (blue): fluid line of tumor reduction showing the drastic fall in tumor size within the first two years of TAE. Gray areas represent 95% confidence interval

**FIGURE 3:**
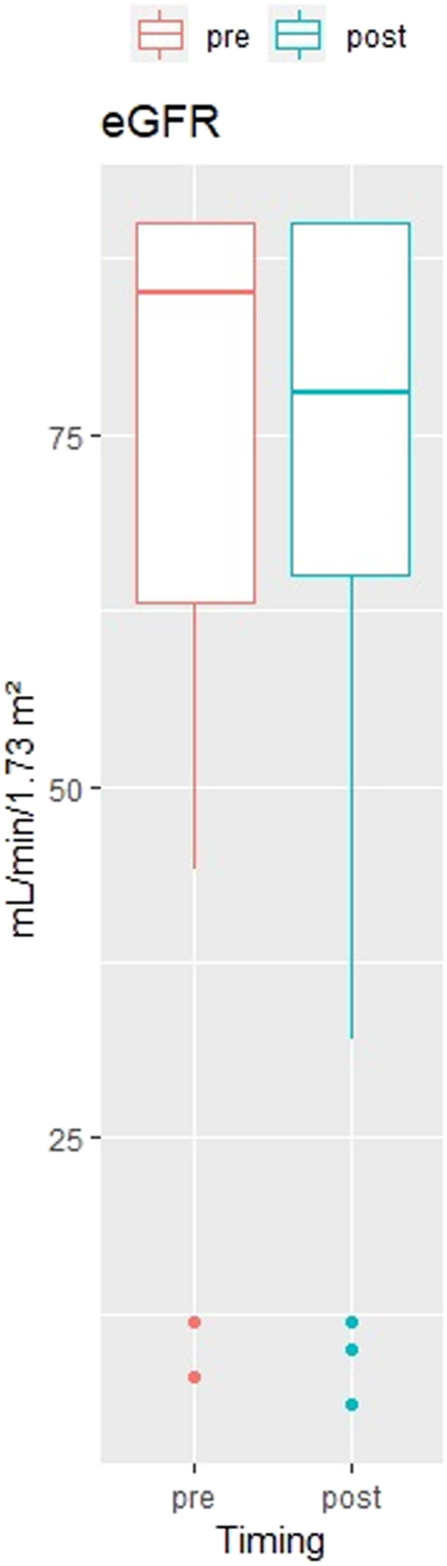
Boxplot of serum creatinine estimated glomerular filtration rate (eGFR) measured in patients pre‐ and post‐embolization (*n* = 49). eGFR levels were missing from nine procedures after embolization and three procedures prior to embolization. There is no significant difference between pre‐ and post‐embolization measurements of eGFR (*p* = 0.61)

The median preoperative tumor size of the 53 tumors in the 49 patients was 6.0 cm [IQR: 4.6–7.0 cm] and was significantly reduced postoperatively to 3.7 cm [2.5–5.2 cm] (*p* < 0.001) during the follow‐up time. The change in tumor size and change in the weighted mean size in the study period is depicted in Figure 2. The median preoperative eGFR was 85 ml/min/1.73 m^2^ [IQR, 63.5–90] and did not change significantly after TAE. The median postoperative eGFR was 80 ml/min/1.73 m^2^ [IQR, 66.5–90], (*p* = 0.89) (Figure 3).

Three deaths, not related to AML, was noted during follow‐up, all within the first 2 years after TAE.

## 5 DISCUSSION

This study demonstrated a significant reduction in median tumor size with a low risk of severe complications requiring surgical reintervention following selective embolization of AML. The majority of procedures was performed in local anesthesia in an outpatient setting, which could be considered an advantage of the procedure compared with surgical options that require general anesthesia and admission to the hospital.

There are several treatment options available for patients with AML that include both active treatments and observational strategies. Asymptomatic, incidentally discovered tumors may be optimal for active surveillance including a regular imaging protocol for radiological assessment of tumor size together with clinical examinations. The main challenge for surveillance is to decide a threshold for intervention. Tumor size is typically used as a target for intervention although a retrospective study of the natural history of AML found no difference in tumor growth rate between tumors larger or smaller than 4 cm.^17^ On the other hand, spontaneous AMLs may rupture and cause severe, and sometimes lethal, retroperitoneal hemorrhage. Studies have shown that 50% of tumors > 4 cm have microaneurysms as an early warning sign of increased risk of rupture.^18^ However, the risk factors for rupture of AML beyond tumor size are not well understood and may be affected by other factors such as anticoagulant treatment, hypertension, physical trauma, pregnancy, and other unknown parameters.^19^ A study by Nason et al speculated that the number needed to treat with prophylactic surgery to prevent one emergency bleeding in AML < 4 cm is 136 and 205 to prevent one blood transfusion, which underlines that an active strategy can be associated with a high risk of overtreatment.^20^ Lastly, regular radiological follow‐up during surveillance raises concerns about the radiation dose given to the patients. This should be considered when counseling the patient, especially younger patients who need long follow‐up. The decision to initiate active surveillance or switch to active therapy during surveillance is a careful assessment of the individual patient, including patient preference. Also, it is controversial if the prophylactic nature of the intervention is well balanced against the morbidity associated with the procedure.

Several active surgical treatment options have been studied over the past decades, and there are currently no recommended gold standard. Open and laparoscopic removal of the tumor with nephron‐sparing surgery is currently the most frequently reported procedure (31% of all AML treatments) for treating AML. Nephron‐sparing surgery may require hospitalization on average between 2 and 9 days.^8^ However, recent studies with robot‐assisted laparoscopy suggest that partial nephrectomy of renal masses may be performed as a same‐day procedure.^21^ In AML, a study by Kara et al. included 54 patients who underwent robot‐assisted laparoscopic surgery. The median follow‐up time was 7 months (range 1–17 months). The mean estimated blood loss was 198 ml (SD ± 194), and postoperative complication rates were 15% (including ileus, pulmonary insufficiency, infections, atrial fibrillation, deep vein thrombosis, and prolonged lymphatic drainage) as well as postoperative transfusion rates of 9.4%.^22^ These results seem comparable with Golan et al. where 40 patients underwent robot‐assisted laparoscopic surgery for AML. No difference in complications was found for those who underwent SAE prior to robot‐assisted laparoscopic surgery compared with those who did not. In that series, 5% experienced a loss of renal function after surgery.^23^ Open surgery has also been investigated. Boorjian et al. studied 58 partial nephrectomies for AML and found a rate of surgical reintervention rate of 12% and no impairment of renal function over 8 years of follow‐up but 23% needed blood transfusion. A total of 3.4% experienced a recurrence of AML.^9^ Overall, open or laparoscopic surgery is safe and with a low risk of recurrence.

Minimally invasive treatments such as tumor ablation using cryotherapy, microwave‐, or radiofrequency ablation are alternative options for the treatment of AML. In Castle et al., radiofrequency ablation was used in tumors ranging from 1.0 to 3.7 cm in 13 patients, with no radiographic evidence of persisting AML at a mean of 21.1 months follow‐up. A total of four (26%) patients experienced postoperative complications including myocardial infarction and pneumonia.^24^ Byrd et al. reported a series of seven patients using laparoscopic cryoablation to treat AMLs up to 7 cm, with an average hospitalization of 3 days. Overall tumor reduction was surprisingly not reported. Two patients experienced complications that included superficial liver laceration and perinephric hematoma.^25^ Zhi‐Yu et al. showed complete ablation of 15 lesions (mean size of 3.4 cm) from 19 treated lesions with microwave therapy during a mean follow‐up time of 10 months. No hospitalization stay was reported, although a colon fistula was observed in 1 patient out of 14 who later underwent surgery. Another patient was observed with abdominal swelling, which later was diagnosed as an infection at the site of ablation, and required drainage.^26^ So far, there is limited evidence for ablation techniques of AML. The main limitation of thermal ablation is tumor size, which should be limited to approximately 3 cm or less for optimal delivery of energy to the tumor.^27^

There has been a great interest in TAE for the treatment of AML, primarily because it can be performed in local anesthesia and in an outpatient setting. Moreover, tumor size is not a limitation. Our study showed a 40% decrease in tumor diameter, which is comparable with the results found in a systematic review of TAE by Murray et al.^28^ The most frequent complication to TAE is PES, ranging from 12.5% in Ramon et al.^29^ findings to 56% of cases presented by Fernandez‐Pello et al.^8^ The incidence of PES was 55% in this study. The incidence of PES varies presumably due to various definitions of PES, as no strict criteria in the current international guidelines exist. Severe complications can also occur after TAE, including renal abscess and thrombosis of the renal artery. Severe complications were reported in 2.9% of the 71 patients in the study by Anis et al., which resulted in complete loss of renal function.^13^ Lastly, TAE may not result in a complete response, which was also demonstrated in our study. Murray et al. reviewed 31 studies that included 524 AML cases and reported a 20% reintervention rate at a mean follow‐up of 39 months, which is comparable with our series where 20.4% underwent re‐embolization.^28^ Limited TAE studies have reported the need for hospitalization. To our knowledge, we report the shortest overall hospitalization stay for both acute and prophylactic embolization procedures. Urbano et al. reported a mean hospitalization of 1.5 days after elective embolization.^30^ It remains unknown which treatment of AML is the most optimal. There is currently no randomized trial that have compared different surgical interventions, and these are much needed. For now, selection of treatment should follow patient preference and assessment of comorbidity. One major disadvantage of TAE is the fact that it cannot be performed in patients with impaired renal function and estimated glomerular filtration less than 35. Also, no histological information is obtained, and renal cancer could potentially be missed. Nonetheless, TAE may be an alternative treatment option in patients who are not optimal candidates for general anesthesia, have previous abdominal surgery or high risk of surgical complications due to other comorbidities.

Medical treatment of AML is also a treatment option for patients with TSC. The mTOR inhibitor Everolimus has been investigated in several studies. In a double‐blind, placebo‐controlled phase 3 trial of TSC patients with AML of at least 3 cm, Everolimus reduced tumor size by more than 50% in 42% of the patients compared with 0% in the placebo group. However, acne‐like skin, stomatitis, and nasopharyngitis are frequent side‐effects that impair compliance. Long‐term follow‐up of the EXIST‐2 trial shows that Everolimus remains efficacious beyond 4 years of treatment without severe morbidity.^7^ In our study, seven patients have prescribed Everolimus after SAE. We did not record the doses taken but did notice that many patients reported compliance problems due to side effects.

This study has strengths and limitations worth mentioning. The strengths include complete and long‐term follow‐up that captured changes in tumor size and the need for re‐embolizations, which has been one of the foremost critiques of TAE. Also, the use of national electronic medical records recorded all readmissions, complications, and long‐term mortality, which is important to describe the long‐term implication of TAE. The main limitation is the small cohort and retrospective nature of the study that did not include a comparator to other surgical or observational interventions. TAE may be used for patients with a better general health, and this may complicate the comparison with studies of other treatments used for AML. A study of observation versus one or more interventions in the prophylactic treatment of AML is warranted.

In conclusion, our results demonstrate that selective arterial embolization of AML significantly reduced the median tumor size with a low number of severe complications and no significant loss of renal function with a long‐term follow‐up. TAE is an alternative treatment option for AML and can be considered in patients who are not optimal candidates for abdominal surgery. The most important drawback of TAE is the need for reintervention that occurs in up to 20%. TAE should be compared with surgical treatment in comparative trials, and there is still limited knowledge about factors that predict an optimal response to TAE, which may primarily be related to the material used for embolization.

## CONFLICTS OF INTERESTS

All authors declare no conflicts of interest.
